# Delineating soil fertility management zones using geostatistics and fuzzy clustering in semi-arid maize systems in India

**DOI:** 10.1007/s10661-025-14608-z

**Published:** 2025-10-21

**Authors:** Pandit Vaibhav Bhagwan, Theerthala Anjaiah, Chitteti Ravali, Makam Uma Devi, Tadikamalla Laxmi Neelima, Darshanoju Srinivasa Chary, Sumanta Chatterjee

**Affiliations:** 1 Department of Soil Science, School of Agriculture, SR University, Ananthasagar, Warangal, Telangana 506371 India; 2 Institute of Soil Health Management, Professor Jayashankar Telangana State Agricultural University, Hyderabad Telangana, Rajendranagar, Hyderabad, Telangana 500030 India; 3 Regional Agricultural Research Station, Polasa, Jagtial, Professor Jayashankar Telangana State Agricultural University, Rajendranagar, Hyderabad, Telangana 500030 India; 4 Department of Agronomy, College of Agriculture, Professor Jayashankar Telangana State Agricultural University, Rajendranagar, Hyderabad, Telangana 500030 India; 5 Department of Mathematics and Statistics, College of Agriculture, Professor Jayashankar Telangana State Agricultural University, Rajendranagar, Hyderabad, Telangana 500030 India; 6 Department of Environmental Science and Technology, University of Maryland, College Park, MD 20742 USA; 7 Department of Biological Sciences, Mississippi State University, Starkville, MS 39759 USA

**Keywords:** Fuzzy C mean clustering, Precision agriculture, Principal component analysis, Spatial variability, Site specific nutrient management, Kriging

## Abstract

This study quantified spatial variability in soil fertility attributes to delineate management zones (MZs) for site-specific nutrient management (SSNM) in a 4-ha maize field in northern Telangana, India. A total of 200 geo-referenced surface (0–15 cm) soil samples were analyzed for pH, electrical conductivity, organic carbon, and available nutrients (e.g., P, K, S, Fe, Mn, Zn, and Cu). Geostatistical analysis using ordinary kriging revealed that spherical models best were the best fit for describing the spatial structure of most parameters, with strong spatial dependence (nugget/sill < 0.25). Principal Component Analysis (PCA) reduced dimensionality, and fuzzy C-means clustering of the principal components delineated three distinct MZs, which were validated by ANOVA. Integration of MZs with targeted yield-based fertilizer recommendation equations enabled differential NPK application, resulting nutrient use efficiency gain equivalent to savings of up to 36 kg N, 39 kg P₂O₅ and 31 kg K₂O ha⁻^1^ in MZ -3. The maize yield increased from 7.27 t ha^−1^ under conventional farmer practices to 7.79 t ha^−1^ in MZ -1, 7.93 t ha^−1^ in MZ-2 and 8.02 t ha^−1^ in MZ -3 with corresponding benefit–cost ratio of 2.54, 2.60 and 2.65. MZ-3 consistently outperformed other zones in yield and economic return, demonstrating the agronomic and economic efficiency of site-specific nutrient management. This work demonstrates the potential of combining geostatistics and fuzzy clustering for optimal nutrient use efficiency and profitability in smallholder maize-based agroecosystems.

## Introduction

The hot and dry semi-arid climate poses significant challenges for sustainable crop management. Soil in these regions exhibit high spatial and temporal variability due to the combined influence of geology, topography, climate, and land use (Venugopal et al., 2024). Rainfall in semi-arid areas are characteristically erratic in intensity and frequency, often resulting in a negative plant–nutrient balance within cropping systems. Continuous land degradation has compromised the long-term viability of many existing cropping systems in these areas (Jinger et al., 2023). Soils with low organic matter contribute to weak soil aggregation and increase susceptibility to physical degradation (Chatterjee et al., 2018). Nutrient use efficiency in such environments is highly variable and typically remains below 25% (Singh et al., 2007). Consequently, farmers often report poor and inconsistent crop responses to applied fertilizers. Precision nutrient management has been shown to improve nutrient use efficiency, thereby enhancing crop yield and biomass production (Chatterjee et al., 2016, 2017).

In maize-based cropping system in semi-arid regions of India, these constraints are intensified by erratic rainfall, high evaporative demand and spatial heterogeneity in soil. This variability results in unequal distribution of nutrient within a field, leading to localized nutrient deficiencies or surpluses. Farmer frequently apply low or high dose of fertilizer, which cause suboptimal yield, wastage of fertilizer and high risk to groundwater pollution through leaching. Given that fertilizer is major component of cost of production and water availability is often a limiting factor, optimizing nutrient use efficiency under such conditions is imperative to ensure both economic viability and environmental sustainability. In this context. Delineation of management zones (MZs) offers a promising approach to address spatial variability within fields (Chatterjee et al., 2018). Management zones are defined as sub field units exhibiting relatively homogeneous soil properties, crop yield potential and fertilizer requirement. The delineated management zones offer site specific nutrient management (SSNM), whereby fertilizer applications can meet the actual nutrient demand of crops within each zone (Davatgar et al., 2012). Numerous scientific investigations across diverse crops and agro-ecological regions have reported that fertilizers application based on management zones can be reduced by 10–30% without compromising crop yield. Scientific evidence for cultivation of maize crop in semi-arid regions of India remain limited. Adoption of MZs could enhance yield stability, nutrient efficiency, and environmental sustainability, but requires robust, locally adapted delineation methods.

To implement precision management effectively, robust methodologies required to assess spatial variability in soil fertility and delineate homogenous MZs within fields (Peralta & Costa, 2013). MZs define sub-field areas with homogenous soil properties, such as nutrient levels, and facilitate SSNM (Chatterjee et al., 2021). However, delineation of such MZs is nuanced by complex and nonlinear interactions among multiple soil and environmental factors. This interaction makes it challenging to accurately characterize within-field variability and define distinct management boundaries (Ramzan et al., 2021; Wang et al., 2023; Zeraatpisheh et al., 2022).

Soil properties exhibit significant spatial variability (Chatterjee et al., 2020). Conventional sampling methods and management practices often fail to capture this spatial heterogeneity. In contrast, precision agriculture (PA) enables targeted interventions by leveraging spatial information on soil variability (Futa et al., 2024; Sharma et al., 2025). Recent advances in geospatial technologies have facilitated collect high-resolution data allow farmer to manage sub-field variability more effectively (Arévalo-Hernández et al., 2024; Bullock et al., 2007; Shukla et al., 2024). Soil nutrient mapping play a critical role in developing and monitoring of site-specific interventions, thereby promoting sustainable input use and productivity gains. Traditional MZs delineation methods include soil surveys, topographic analysis, yield mapping, and nutrient indices (Chatterjee et al., 2021; Kumar et al., 2023; Shashikumar et al., 2023).

In recent decades, soil scientists have increasingly adopted geostatistical techniques to characterize spatial variability in soil properties across various spatial scales (Al Zihad et al., 2023; Chatterjee et al., 2015; Wickramasingha & Weerasinghe, 2024). Geostatistics provides robust tools for analyzing and modeling spatial dependence though semivariogram analysis, which quantifies the relationship between variance in observations and their spatial separation (Goovaerts, 1998; Muniammal Vediappan et al., 2025). In parallel, fuzzy clustering approaches, such as fuzzy C-means (FCM), have been extensively used for delineation MZs by capturing the continuous nature of soil variability and avoid rigid class boundaries (Al Zihad et al., 2023; Guastaferro et al., 2010; Muniammal Vediappan et al., 2025; Zhao et al., 2023).

FCM assigns a membership value to each observation, indicating its degree of association with multiple clusters. This probabilistic framework provide a more realistic representation of soil heterogeneity (Bezdek, 1981; Venugopal et al., 2024; Yuan et al., 2022). However, isolating the contribution of individual soil properties to overall soil quality remains challenging, particularly when establishing sharp boundaries between MZs. To address this, dimensionality reduction techniques like Principal Component Analysis (PCA) are often integrated with clustering to reduce multicollinearity and emphasize dominant variance structures (Salem et al., 2024; Zeraatpisheh et al., 2022). The combination of PCA and FCM has proven effective for MZs delineation in various agroecosystems (Kumar et al., 2023; Neupane et al., 2024; Shukla et al., 2024).

Understanding and managing spatial heterogeneity is essential for efficient input use in PA. Given the strong spatial variability in soil properties, field-level recommendations must be based on detailed spatial analyses. Accordingly, this study aims to (1) assess the spatial variability of soil properties using geostatistical methods, (2) delineate management zones through principal component analysis and fuzzy-C clustering clustering, and (3) evaluate the effectiveness of the MZs in implementing site specific nutrient management in maize-based systems of northern Telangana, India.

## Materials and methods

### Study area

**Fig. 1:**
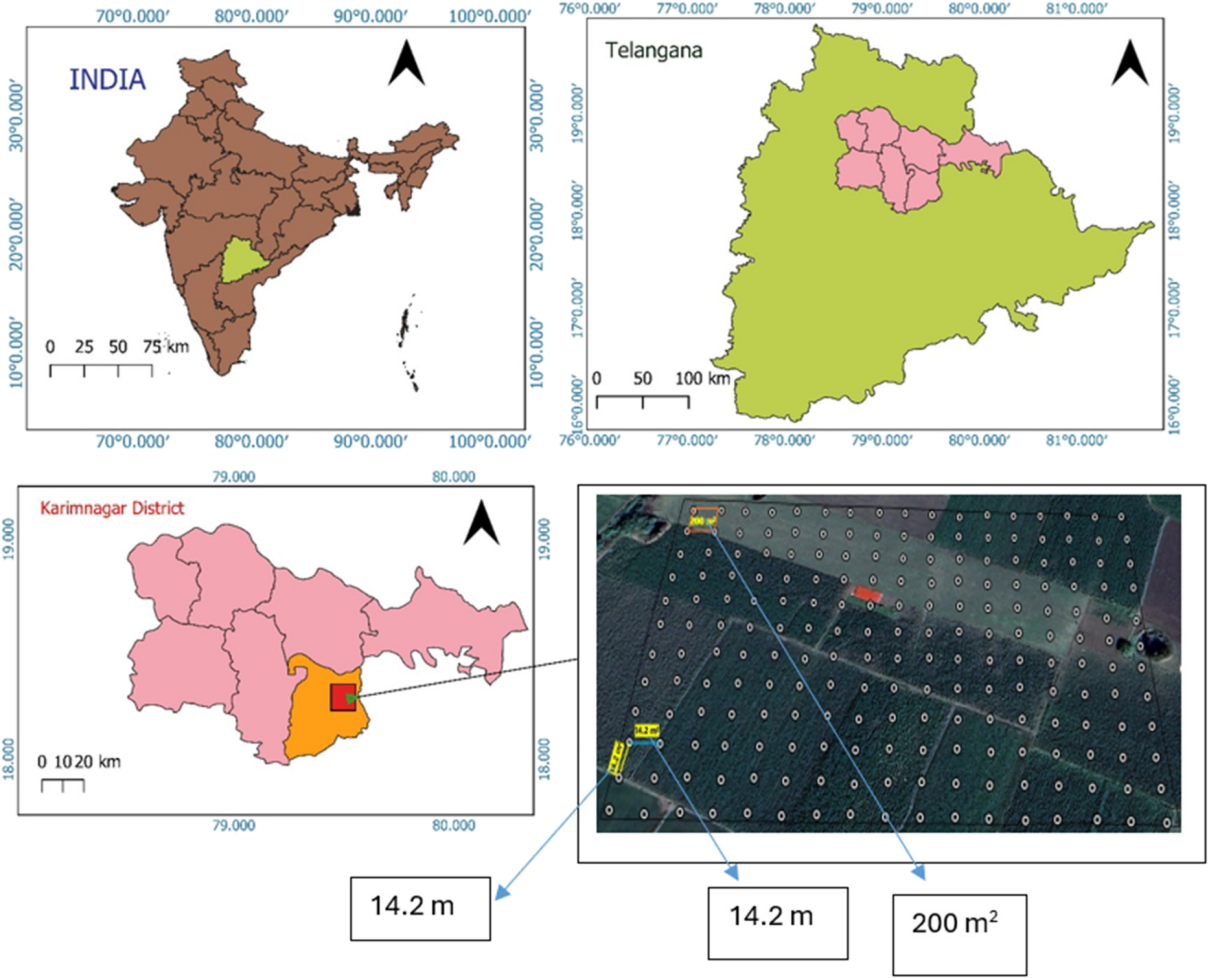
Study site and soil-sampling points in Bogumpadu village, Ellanthakuntha Mandal, Karimnagar district, Northern region of Telangana, India

The study was conducted on a 4-ha maize field located in Bogumpadu village, Ellanthakuntha Mandal, Karimnagar district, located in northern Telangana, India (18.3043°N to 18.3029°N, 79.5490°E to 79.5511°E) (Fig. 1). The site lies within the Deccan Plateau agro-climatic region, classified as a hot, moist, semi-arid subregion with a growing period of 120–150 days (Panjala et al., 2024). The climate is characterized by hot summers, cool winters, and average an annual rainfall of 867 to 1189 mm, of which nearly 86% of rainfall occur during the southwest monsoon (June–September) and the remainder during the northeast monsoon (December–January). Summer maximum temperatures range from 31–39 °C in summer (March–June), while winter minimums range between 14–25 °C (Chanapathi et al., 2020; Tapas et al., 2022; https://www.pjtsau.edu.in, 2021–22).

The soil is deep black, calcareous and fine-textured, with a neutral to strongly alkaline in pH, and a medium to strong subangular blocky structure. According to USDA soil taxonomy, the soil is classified as very fine, smectitic, hyperthermic, Typic Chromusterts (Bhattacharyya et al., 2015; Kumar et al., 1994). The dominant cropping system is cotton in kharif (the monsoon season, June-October) followed by maize in rabi (the post monsoon/winter season, November-March). Recommended fertilizer doses are 150:60:60 kg N:P₂O₅:K₂O ha⁻^1^ for kharif cotton and 250:40:40 kg N:P₂O₅:K₂O ha⁻^1^ for rabi maize (https://www.pjtsau.edu.in, 2021–22). Irrigation is primarily supplied through groundwater.

### Collection and analysis of soil

In 2020, a total of 200 composite topsoil samples (0–15 cm depth) were collected from the study area on a 14.2 m × 14.2 m grid using stainless steel auger, with geolocations recorded by Garmin eTrex handheld GPS device (Fig. 1). At each grid, four subsamples were collected around the grid center and composited into single representative soil sample of approximately 500 g. Each composites samples were air-dried, homogenized, and crushed using a hardwood mallet. The soil was then sieved through a 2 mm mesh for general analysis, while a portion was further sieved to 0.2 mm for organic carbon determination. Soil pH and electrical conductivity (EC) were measured using potentiometric and conductometric methods with a digital pH meter (Thermo Fisher Scientific Orion Star A211, USA) and an EC meter (Thermo Fisher Scientific Orion Star A212, USA), respectively (Mylavarapu et al., 2020). Soil organic carbon (SOC) was determined via wet oxidation using potassium dichromate (Walkley & Black, 1934). Available nitrogen (N) was estimated using the alkaline KMnO₄ method (Subbiah & Asija, 1956) with a Kel Plus Nitrogen Analyzer (Pelican Equipments, India). Available phosphorus (P₂O₅) was extracted with 0.5 M sodium bicarbonate (pH 8.5) following Olsen (1954) and quantified with double beam UV–Visible Spectrophotometer (Shimadzu UV-1800, Japan), while available potassium (K₂O) was determined using 1 N ammonium acetate (Hanway, 1952) and measured with a Flame Photometer (Elico CL 378, India). Available sulphur was analyzed using the turbidimetric method (Chesnin & Yien, 1951) with a double beam UV–Visible Spectrophotometer (Shimadzu UV-1800, Japan). Micronutrients (Fe, Mn, Zn, Cu) were extracted with 0.005 M DTPA solution (Lindsay & Norvell, 1978) and quantified using Inductively Coupled Plasma Optical Emission Spectroscopy (ICP-OES, PerkinElmer Optima 8000, USA).

### Statistical analysis

Descriptive statistics, including minimum, maximum, mean, standard deviation (SD), coefficient of variation (CV), skewness, and kurtosis, were computed for all measured soil properties. Pearson’s correlation coefficients were calculated to assess relationships among soil parameters. All statistical analyses were performed using XLSTAT 2020 and R (version 4.3.1).

### Geostatistical analysis

**Fig. 2:**
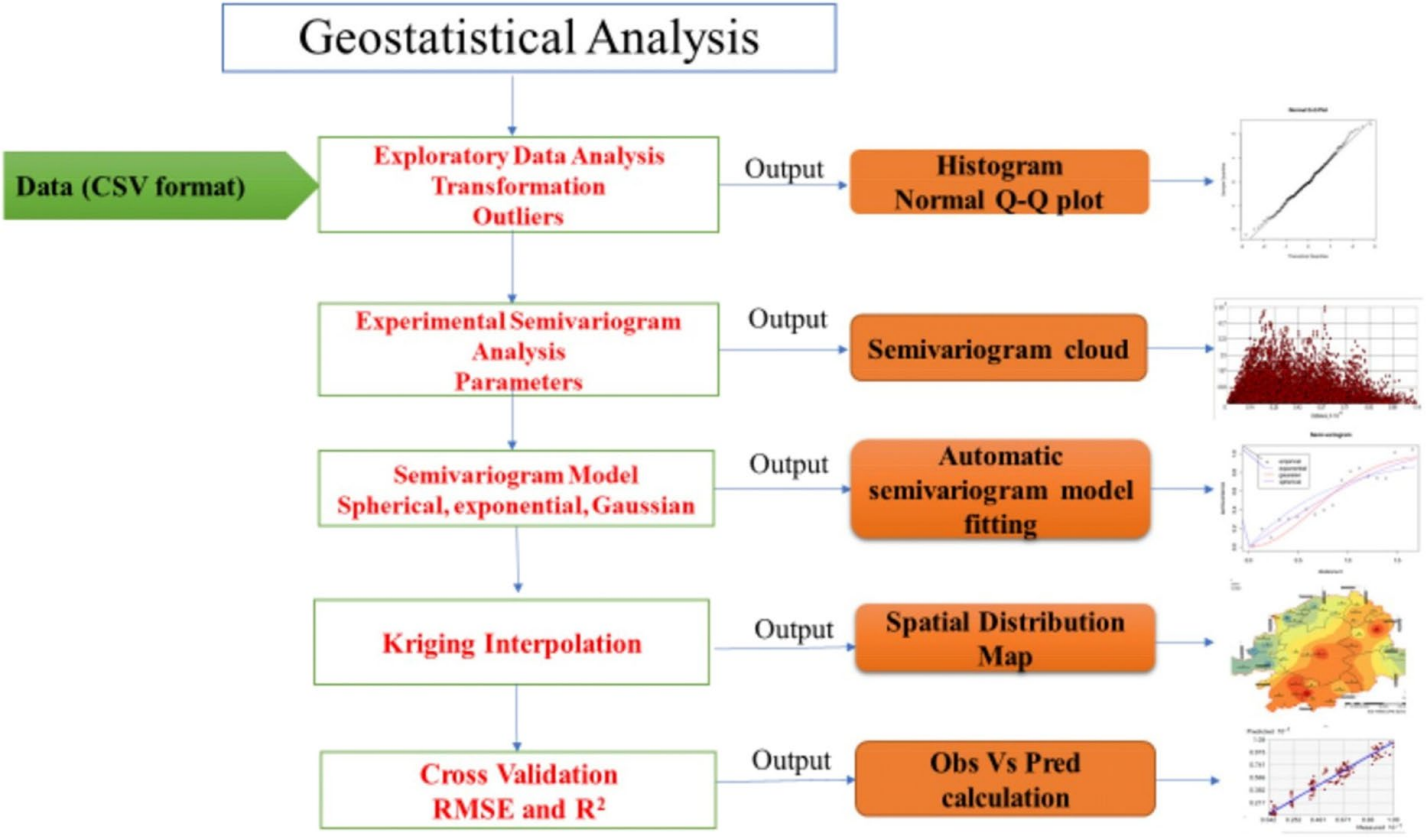
Flowchart of methodology of geostatistical analysis

Geostatistical analysis was performed using ArcGIS 10.8 (ESRI) software to evaluate the spatial variability of soil properties (Shahinzadeh et al., 2022; Webster & Oliver, 2007). Prior to analysis, data normality was assessed using Q-Q plots. Spatial dependence was quantified using semivariograms, which describe the similarity between paired observations as a function of distance (h) (Fig. 2). The empirical semivariance γ̂(h) was calculated as follows (De Caires et al., 2025; Goovaerts, 1998; Selmy et al., 2022):

1 $$\widehat\gamma\left(h\right)=\frac1{2N(h)}\sum\nolimits_{i=1}^{N(h)}\left\lfloor Z\left({\text{x}}_{\left(\text{i}\right)}\right)-Z{(x_{\left(i\right)}+h)}^2\right\rfloor$$

where, N(h) represents the number of data pairs in a specified distance and direction, $Z\left({\text{x}}_{\left(\text{i}\right)}\right)$ represent the value of the variable at the position ${\text{x}}_{\left(\text{i}\right)}$, $Z{({x}_{\left(i\right)}+h)}$ represent the value of the variable at a distance of h from position${\text{x}}_{\left(\text{i}\right)}$.

The above semivariogram equation fitted with standard model to calculated spatial variation parameters: nugget, sill, and range. Semivariograms were fitted using standard models—spherical, exponential, and Gaussian, and their performance evaluated based on cross-validation metrics: normalized root means square error (nRMSE) and coefficient of determination (R^2^) (De Caires et al., 2025). The best-fitting model for each soil parameter was subsequently used for spatial interpolation using ordinary kriging (Arévalo-Hernández et al., 2024). Model performance was assessed using the following equations (Jena et al., 2022; Peter-Jerome et al., 2022):

2 $$\text{nRMSE }=\sqrt{\frac{1}{n}\sum\nolimits_{i=1}^{N}[Z\left({x}_{\left(i\right)}\right)-\overline{Z }\left({x}_{\left(i\right)}\right){]}^{2}}\times \frac{100}{N}$$

3 $${\text{R}}^{2} = 1 - {\left(\frac{\sum\nolimits_{i=1}^{n}\left({M}_{i}-\overline{M }\right)\left({P}_{i}-\overline{P }\right)}{\sqrt{\sum\nolimits_{i=1}^{n}\left({M}_{i}-\overline{M }\right)}\sqrt{\sum_{i=1}^{n}\left({p}_{i}-\overline{P }\right)}}\right)}^{2}$$

where Z(Xi) represents the observed value at position ${x}_{\left(i\right)},$ $\overline{Z }\left({x}_{\left(i\right)}\right)$ represent the predicted value at the position ${x}_{\left(i\right)},$ n is the total number of data, and N is the mean of the observed value. Interpolation accuracy was classified based on nRMSE thresholds: < 20% (good), 20–30% (fair), and > 30% (poor) (Jiang et al., 2021; Zeraatpisheh et al., 2020).

Geostatistics is a key tool for quantifying spatial variability, selecting appropriate models for soil properties, and generating predictive maps (De Caires et al., 2025). The workflow (Fig. 2) includes: (i) data preparation, where soil data with coordinates and measured variables are organized for GIS analysis; (ii) exploratory data analysis (EDA), involving descriptive statistics, plots, outlier detection, and transformations to stabilize variance; (iii) experimental semivariogram analysis, which quantifies spatial dependence among observations through semivariance versus distance, and provides three key parameters such as nugget, sill, and range; (iv) model fitting, where theoretical models (spherical, exponential, Gaussian) are fitted to the semivariogram, and the best model selected using cross-validation statistics (nRMSE, ME, R^2^); (v) mapping, where kriging produces continuous spatial prediction surfaces; and (vi) cross-validation, where predicted and observed values are compared to assess model reliability.

### Principal component analysis (PCA)

**Fig. 3:**
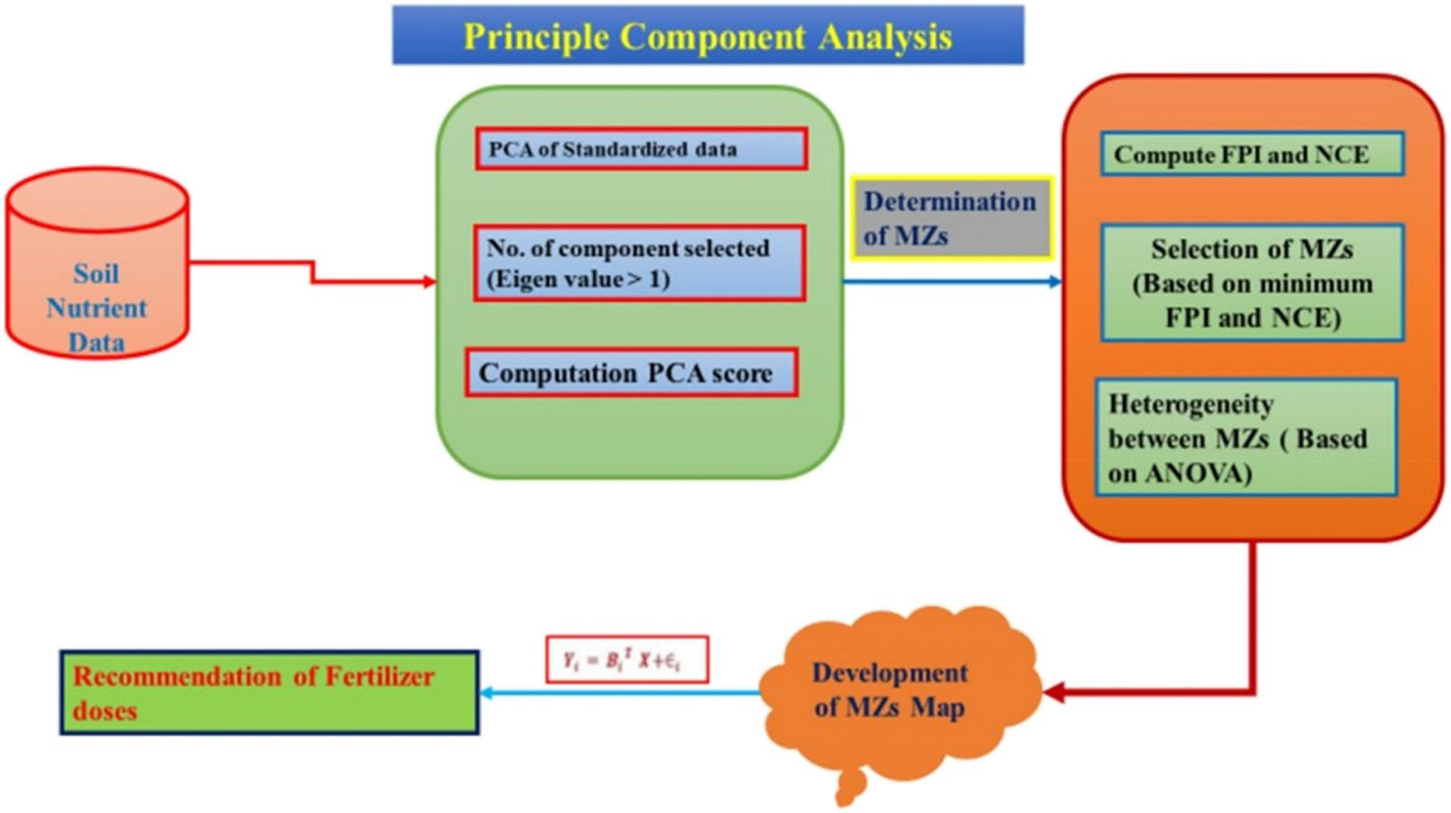
Schematic representation of the methodology of principal component analysis (PCA) and management zones (MZs) for fertilizer recommendation in study area. (FPI: Fuzzy Performance Index and NCE: Normalized Classification Entropy)

Principal Component Analysis (PCA) is a multivariate technique used to reduce data dimensionality and extract underlying patterns from complex datasets (Ramzan et al., 2021) (Fig. 3). This is achieved by rotating the coordinate system to maximize variance along new orthogonal axes, known as principal components. The first few components capture most of the total variance, with each successive component explaining progressively less. In this study, PCA was performed using the covariance matrix of all measured soil parameters, rather than their correlation matrix. Components with eigenvalues ≥ 1 were retained for further analysis and subsequently used for delineating MZs. Factor loadings were examined to identify which soil properties contributed most to each component, thereby facilitating the interpretation of spatial variability (González-Fernández et al., 2019; Shashikumar et al., 2023; Shukla et al., 2024). PCA and exploratory data analysis was performed in R (version 4.5.0) using the packages readxl (for data import), dplyr (for data preprocessing), ggplot2 (for plotting), factoextra (for PCA visualization), FactoMineR (for multivariate analysis), and psych (for supplementary statistical analysis).

### Fuzzy C-means cluster algorithm analysis

The Fuzzy C-Means (FCM) clustering algorithm was employed to delineate homogeneous sub-regions or MZs. Unlike hard clustering methods, FCM allows each data point to belong to multiple clusters with varying degrees of membership, reducing sensitivity to outliers and better capturing transitional boundaries (Shukla et al., 2024; Srinivasan et al., 2022). In this study, the number of clusters varied from a minimum of three to a maximum of eight to identify the optimal zoning configuration (Zhao et al., 2023). The algorithm initiates by randomly assigning cluster centers and iteratively updates membership values and cluster centroids based on the Euclidean distance between data points and cluster means, assuming equal variance and statistical independence. Management Zone Analyst (MZA v.1.0.0) software was used to perform the clustering with the following parameters: maximum iterations = 300, convergence threshold = 0.0001, cluster range = 3 to 8, and fuzziness exponent = 1.5 (Fridgen et al., 2004; Kumar et al., 2023; Minasny & McBratney, 2006; Tripathi et al., 2015; Venugopal et al., 2024). The optimal number of clusters was determined using the Fuzzy Performance Index (FPI) and Normalized Classification Entropy (NCE), calculated using the following equations (Bezdek, 1981; Boydell & McBratney, 2002; Mcbratney & Moore, 1985; Shashikumar et al., 2023):

4 $$\text{FPI}=1-\frac{\text{C}}{\text{C}-1} [1- \frac{ \sum_{\text{i}=1}^{\text{c}}\sum_{\text{k}=1}^{\text{n}}{\left({\text{u}}_{ik}\right)}^{2} }{\text{n}}]$$

5 $$\text{NCE}= \frac{\text{n}}{\text{n}-\text{C}}[\frac{\sum_{\text{k}=1}^{\text{n}}\sum_{\text{i}=1}^{\text{c}}\left({\text{u}}_{ik}{\text{log}}_{a}({\text{u}}_{ik}\right)}{\text{n}}]$$

where C is the number of clusters; n is the number of observations; ${\text{u}}_{ik}$ is the element ik of the fuzzy membership and log_a_ is the natural logarithm.

**Fig. 4:**
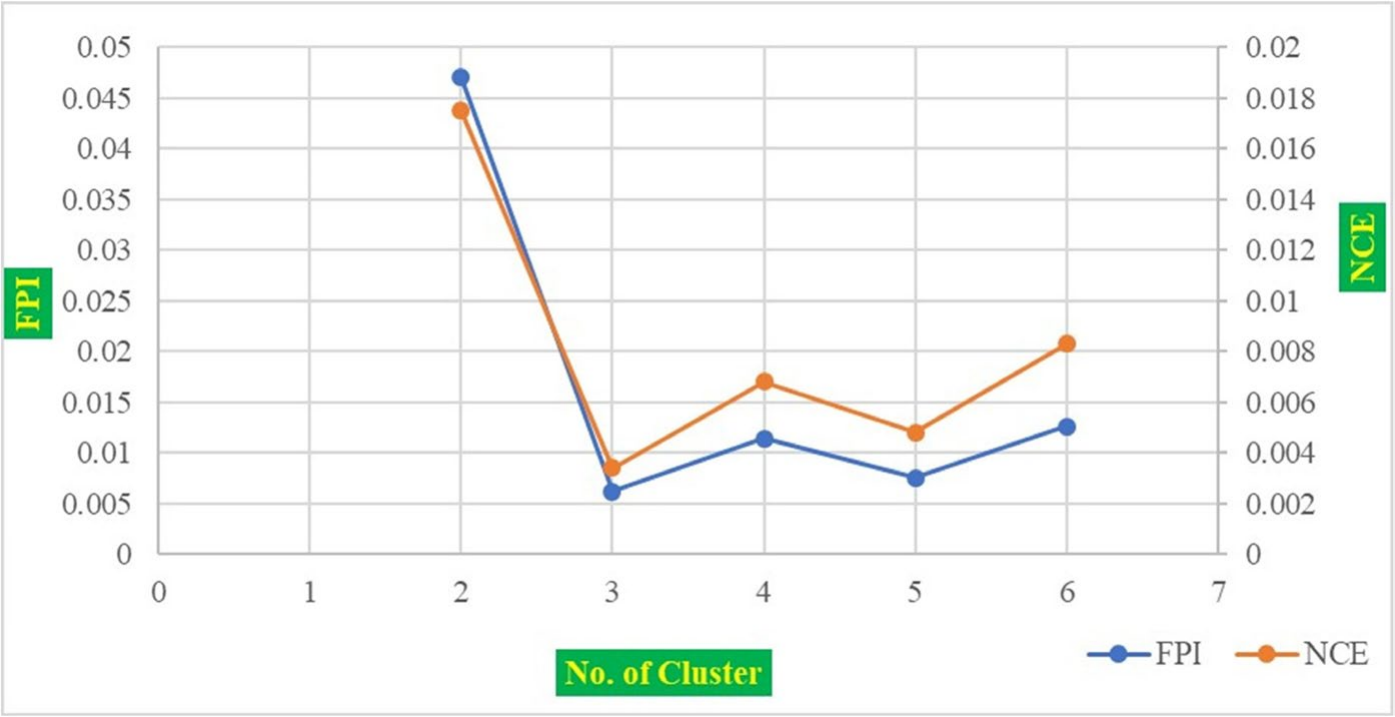
Fuzzy performance index (FPI) and normalized classification entropy (NCE) for identifying the optimum management zone clusters

FPI and NCE are widely used indices to determine the optimal number of clusters in FCM clustering. Lower values of FPI and NCE indicate better-defined clusters with minimal overlap. An optimal cluster solution with the lowest FPI and NCE values was selected for final MZs delineation (Fig. 4) (Dad & Shafiq, 2021; Fridgen et al., 2004; Venugopal et al., 2024). To evaluate differences in soil properties among the MZs, a one-way ANOVA was performed using SPSS (version 26.0) software (Peter-Jerome et al., 2022). The final MZ maps were generated in ArcGIS 10.8 (Verma et al., 2021).

## Results and discussion

### Descriptive statistics of soils parameters

Descriptive statistics for soil parameters are presented in Table 1. Soil pH ranged from 7.07 to 8.52 with a mean of 7.99, indicating a neutral to slightly alkaline reaction The coefficient of variation (CV) was low (2.88%), suggesting minimal variability, consistent with earlier studies (Awe et al., 2025; Jena et al., 2022; Verma et al., 2021; Zhang et al., 2024). This stability is attributed to the soil deviations from basic parent materials (Alnaimy et al., 2023; Narsaiah et al., 2018). The logarithmic nature of pH, coupled with the buffering capacity of these soils, further explains the limited variability in pH despite diverse agricultural practices in the region.

**Table 1:** Descriptive statistics of soil samples collected from the study area

Parameters	Min	Max	Range	Skewness	Kurtosis	SD	Mean	CV (%)
pH	7.07	8.52	1.45	0.19	0.33	0.23	7.99	2.88
EC (dS m^−1^)	0.091	0.199	0.108	0.51	−0.52	0.03	0.14	19.05
OC (g kg^−1^)	2.3	5.6	3.3	−0.28	−0.94	0.07	3.7	18.92
Available N (kg ha^−1^)	70	154	84	−0.20	−0.67	22	111	19.55
Available P_2_O_5_ (kg P_2_O_5_ ha^−1^)	81	145	64	0.14	−0.52	16	108	14.51
Available K_2_O (kg K_2_O ha^−1^)	327	524	197	0.31	−1.15	54	420	12.74
Available S (mg kg^−1^)	10	31	21	0.26	−0.87	6	19	29.42
Fe (mg kg^−1^)	3.2	20.6	17	0.09	−1.04	4.9	11.1	44.14
Mn (mg kg^−1^)	1.9	12.8	11	0.19	−1.09	3.3	7.1	46.48
Zn (mg kg^−1^)	1.4	6.3	4.9	0.42	−0.92	1.5	3.4	44.12
Cu (mg kg^−1^)	2.2	8.9	6.7	0.55	−0.85	1.9	4.7	40.43

Soil Samples collected, *N* = 200

Electrical conductivity (EC) values ranged between 0.091 to 0.199 dS m^−1^ with a mean of 0.14 dS m^−1^ showed low variability as well, with a CV of 19.05%, skewness of 0.51, and kurtosis of –0.52. This can be attributed to the use of non-saline groundwater for irrigation, as reported in other parts of Telangana (Narsaiah et al., 2018; Ramulu & Kamalakar, 2022; Shalini et al., 2022; Vilakar et al., n.d.).

The mean soil organic carbon (SOC) content was 0.37% ranging from 0.23 to 0.56%, which is considered low. These findings align with previous studies reporting low SOC levels (0.5%) across various districts in Telangana (Narsaiah et al., 2018; Shalini et al., 2022; Vilakar et al., n.d.). Low SOC is likely due to limited organic manure application, high decomposition rates under elevated temperatures, minimal nitrogen fertilizer use, and the common practice of not retaining crop residues (Chatterjee et al., 2018; Nogiya et al., 2024; Tripathi et al., 2015; Venugopal et al., 2024).

SOC, available nitrogen, phosphorus (P₂O₅), and potassium (K₂O) all showed low spatial variability (CV < 25%), with CV values ranging from 12.74% to 19.55%, consistent with prior findings from similar agroecosystems (Behera et al., 2022; Belay et al., 2024; Qinghuo et al., 2019; Tagore et al., 2014; Wickramasingha & Weerasinghe, 2024). Available nitrogen ranged from 70 to 154 kg ha⁻^1^ with a mean of 111 kg ha⁻^1^. Its low content and variability may be due to nitrogen losses through leaching and rapid mineralization (Verma et al., 2021; Moharana et al., 2020), along with suboptimal fertilizer application by farmers likely explain theses pattern (Nogiya et al., 2024; Reza et al., 2017; Tripathi et al., 2015).

Available P₂O₅ and K₂O levels were high, ranging from 81–145 kg ha⁻^1^ (mean: 108 kg ha⁻^1^) and 327–524 kg ha⁻^1^ (mean: 420 kg ha⁻^1^), respectively., contribute to accumulation of recalcitrant calcium-phosphate complexes under continuous P fertilizer application without soil testing (Himabindu et al., 2022; M et al., 2024; Malavath & Mani, 2014; Ramulu & Kamalakar, 2022; Sathish et al., 2018; Sravani et al., 2023; Vilakar et al., 2021). High potassium levels are likely due to mineral weathering, potassium release from non-exchangeable sites in 2:1 clay, organic residue decomposition, and capillary rise of K^+^ in groundwater (Manoj et al., 2024; Ramulu & Reddy, 2018; Ravi et al., 2017; Sathish Kumar et al., 2023). Available sulphur exhibited moderate variability (CV = 29.42%) and ranged from 10 to 31 mg kg⁻^1^, with a mean of 19.0 mg kg⁻^1^. These concentration are considered high (Shukla et al., 2017), and may reflect variability in organic recycling, fertilizer inputs, and crop management practices (Aliyu et al., 2020; Behera et al., 2018; Tagore et al., 2014).

Micronutrients including Fe, Mn, Zn, and Cu showed moderate variability (CV between 25–75%, per Wilding’s classification). The mean values were 11.1 mg kg⁻^1^ (Fe), 7.1 mg kg⁻^1^ (Mn), 3.4 mg kg⁻^1^ (Zn), and 4.7 mg kg⁻^1^ (Cu), aligning with prior studies in Telangana (Ramulu & Reddy, 2018). Spatial variability in micronutrients ranging from 44% (Mn) to 110% (Zn) was also reported by Barman et al. (2021) and Dad and Shafiq (2021) and is influenced by management practices such as fertilizer use and crop rotation (Nogiya et al., 2024; Shukla et al., 2017; Singh et al., 2024).

Overall, the observed variability in soil nutrient distribution highlighted the need of delineation of site-specific management zones to enhance input-use efficiency and maize productivity in the study area (Chatterjee et al., 2021; Moharana et al., 2023; Tripathi et al., 2015).

### Geostatistical analysis

**Fig. 5:**
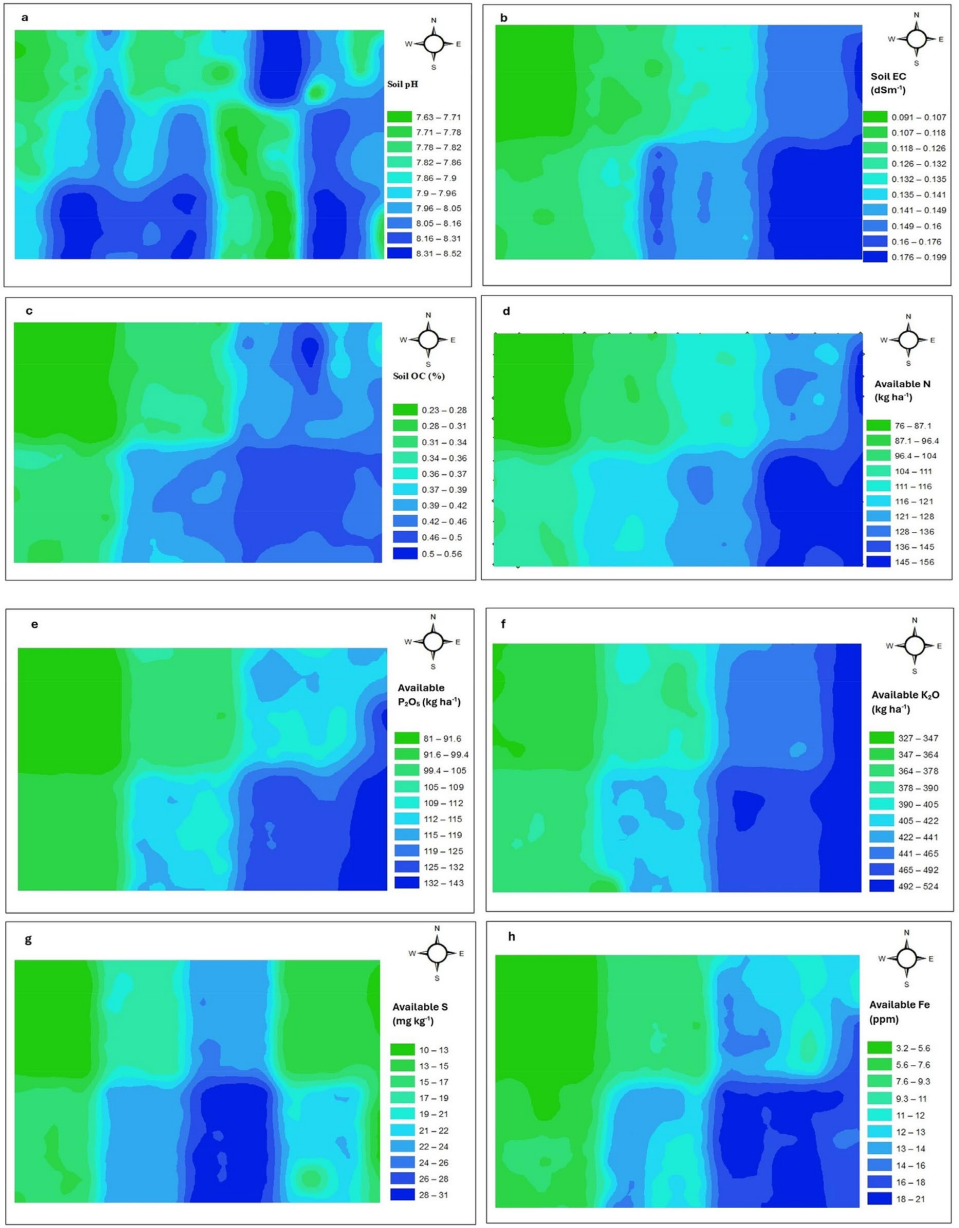

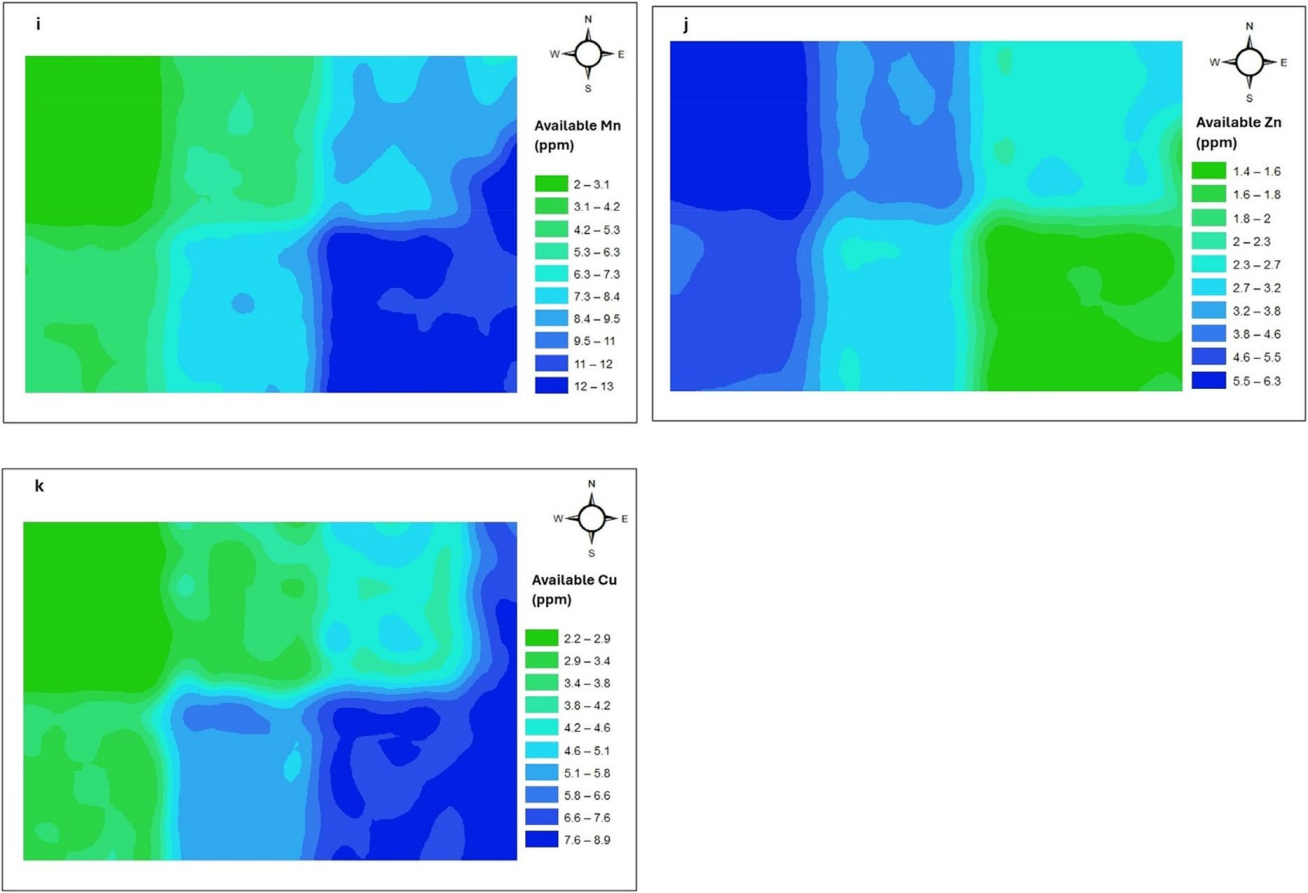
(**a**) Spatial distribution map of pH, (**b**) Spatial distribution map of EC, (**c**) Spatial distribution map of organic carbon, (**d**) Spatial distribution map of available N, (**e**) Spatial distribution map of available P_2_O_5_, (**f**) Spatial distribution map of available K_2_O, (**g**) Spatial distribution map of available S, (**h**) Spatial distribution map of available Fe, (**i**) Spatial distribution map of available Mn, (**j**) Spatial distribution map of available Zn, (**k**) Spatial distribution map of available Cu

Semivariogram model parameters for various soil properties are presented in Table 2. Among the spherical, exponential, and Gaussian model tested, the best-fitted model for each parameter was selected based on the lowest nRMSE and highest R^2^ values. This approach follows previous studies employing ordinary kriging (OK) for spatial interpolation (Arévalo-Hernández et al., 2024; Awe et al., 2025; Chatterjee et al., 2021; Neupane et al., 2024; Shahinzadeh et al., 2022). Spatial distribution maps for the soil properties are shown in Fig. 5(a–k).

**Table 2:** Semivariogram analysis of soil samples (N = 200) collected from the study area

	Trans-formation	Model	Nugget	Partial Sill	Sill	Nugget/sill (%)	Range (m)	Spatial dependence	nRMSE	R^2^
pH	Log	Spherical	0	0.00068	0.00068	0	49.79	Strong	1.38	0.56
EC (dS m ^−1^)	Log	Exponential	0	0.00488	0.00488	0	32.25	Strong	5.71	0.71
Organic carbon (g kg^−1^)	Log	Exponential	0	0.0122	0.0122	0	70.59	Strong	0.68	0.61
Available N (kg N ha^−1^)	None	Spherical	0	54.53	54.53	0	38.95	Strong	7.29	0.61
Available P_2_O_5_ (kg P_2_O_5_ ha^−1^)	Log	Spherical	0	0.003	0.003	0	42.01	Strong	3.21	0.90
Available K_2_O (kg K_2_O ha^−1^)	Log	Spherical	0	0.0019	0.0019	0	40.07	Strong	2.98	0.90
Available S (mg kg^−1^)	Log	Spherical	0	0.084	0.084	0	113.16	Strong	9.42	0.81
Available Fe (mg kg^−1^)	None	Spherical	0	4.86	4.86	0	44.04	Strong	11.44	0.86
Available Mn (mg kg^−1^)	Log	Spherical	0	0.045	0.045	0	55.23	Strong	10.56	0.90
Available Zn (mg kg^−1^)	Log	Gaussian	0.0025	0.0353	0.0378	6.61	40.45	Strong	8.53	0.92
Available Cu (mg kg^−1^)	Log	Gaussian	0	0.027	0.027	0	31.35	Strong	9.79	0.88

**Fig. 6:**
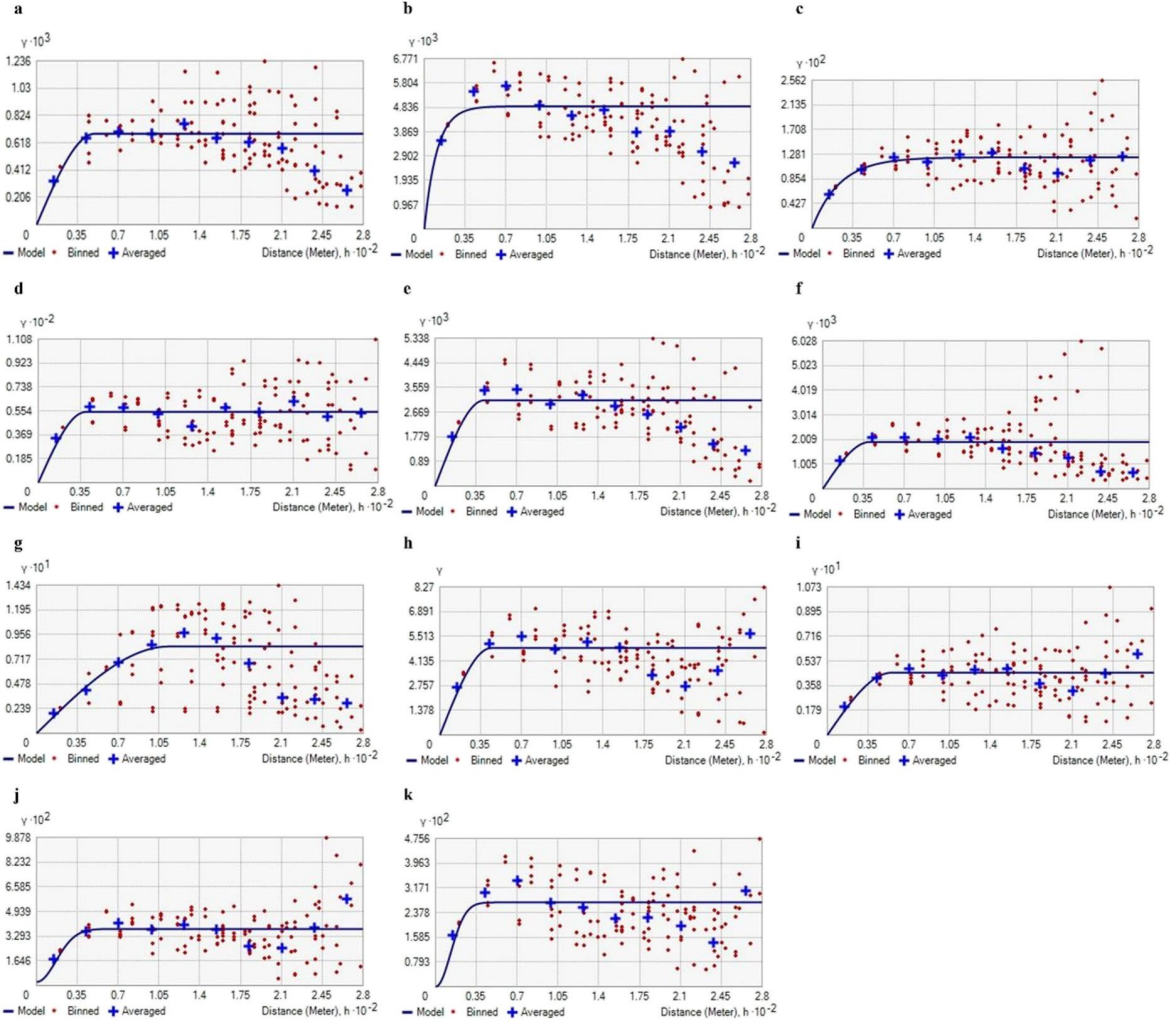
(**a**) Best fitted semivariogram model of pH, (**b**) best fitted semivariogram model of EC, (**c**) best fitted semivariogram model of organic carbon, (**d**) best fitted semivariogram model of available N, (**e**) Best fitted semivariogram model of available P_2_O_5_, (**f**) best fitted semivariogram model of available K_2_O, (**g**) best fitted semivariogram model of available S, (**h**) best fitted semivariogram model of available Fe, (**i**) best fitted semivariogram model of available Mn, (**j**) best fitted semivariogram model of available Zn, (**k**) best fitted semivariogram model of available Cu

The spherical model provided the best fit for the semivariograms of soil pH, available nitrogen, phosphorus (P₂O₅), potassium (K₂O), sulphur, iron (Fe), and manganese (Mn) (Fig. 6a, d–i), in line with earlier reports identifying the spherical model as most appropriate for these parameters (Bogunovic et al., 2017; Moharana et al., 2023; Neupane et al., 2024; Nourzadeh et al., 2012; Singh et al., 2024; Tamburi et al., 2020; Tripathi et al., 2015; Vasu et al., 2021; Venugopal et al., 2024; Xin-Zhong et al., 2009). Soil EC and SOC were best described by the exponential model (Fig. 6b, c), consistent with previous findings (Barman et al., 2021; Bhunia et al., 2018; Chatterjee et al., 2021; Duan et al., 2020; Liu et al., 2014; Muniammal Vediappan et al., 2025; Salem et al., 2024; Sharma & Sood, 2020; Usowicz & Lipiec, 2017; Yeneneh et al., 2022). Zinc and copper followed Gaussian model patterns (Fig. 6j, k), similar to findings by Nogiya et al. (2024) and Gökmen et al. (2023).

The semivariogram parameters—nugget, sill, and range capture the sources of spatial variability, whether intrinsic (e.g., parent material) or extrinsic (e.g., management practices) (Behera et al., 2015; Moharana et al., 2020; Rahul et al., 2019; Salem et al., 2024; Shukla et al., 2024). Nugget values, indicating micro-variability and measurement error, ranged from 0 to 0.0025. Except Zn all parameters exhibited a zero-nugget effects, suggesting negligible unexplained variability (Shahinzadeh et al., 2022; Verma et al., 2021). The sill represents the point at which the semivariance stabilizes and approximates the variance of the sampled population, ranged from 0.00068 (pH) to 54.53 (available nitrogen), consistent with geostatistical theory (Benedito Mendes Brito et al., 2018; Mulla, 2012; Muniammal Vediappan et al., 2025; Nasiri Dehsorkhi et al., 2025).

According to Cambardella et al. (1994), nugget-to-sill ratios < 0.25 indicate strong spatial dependence (driven by intrinsic factors), 0.25–0.75 indicate moderate dependence (mixed control), and > 0.75 suggest weak dependence (external influences). All soil parameters in this study exhibited strong spatial dependence, implying control by inherent soil-forming factors such as parent material, topography, and climate (Behera et al., 2018; Soropa et al., 2023; Zhang et al., 2024). These findings align with previous studies reporting strong spatial dependence for soil pH, SOC, nitrogen, phosphorus, and micronutrients (El Hamzaoui & El Baghdadi, 2021; Ramzan et al., 2021; Selmy et al., 2022; Sharma & Sood, 2020; Verma et al., 2018, 2021; Yeneneh et al., 2022; Xin-Zhong et al., 2009).

The semivariogram range defined the spatial scale over which samples remain correlated. In this study, most soil parameters (except SOC and sulphur) exhibited spatial correlation ranges of 30—55 m, indicating sample values are spatially correlated within this distance and approaches randomness beyond it (Kumar et al., 2022, 2023; Dad & Shafiq, 2021; Moharana et al., 2020; Neupane et al., 2024; Reza et al., 2017; Venugopal et al., 2024).

Figure 5(a–k) illustrates the interpolated spatial distribution of all soil properties. Most nutrients (except Zn) were higher in the southeast, east, and southern zones, while lower levels were observed in the northern and northwestern areas. Concentration of pH, EC, nitrogen, phosphorus, potassium, sulphur, Fe, Mn, and Cu increased from northeast to southeast, from north to south, and from west to east. In contrast, Zn showed inverse trend, these contrasting spatial pattern likely due to differences in parent material, irrigation practices, fertilizer use, and cropping patterns in this region in India.

Such spatially explicit information provides a scientific basis for SSNM. Identifying nutrient-rich and deficient zones enables the design of variable-rate fertilization strategies, which can enhance nutrient-use efficiency, reduce environmental losses, and improve maize productivity in a sustainable manner.

### Principal components analysis

Correlation analysis among soil properties revealed significant interrelationships (Table 3). To reduce data dimensionality, multicollinearity, and identify key patterns in the dataset, PCA was performed (Kumar et al., 2023; Srinivasan et al., 2022; Xin-Zhong et al., 2009). PCA generates a number of components equal to the number of input variables. In this study, 11 principal components (PCs) were generated, but only the first five with eigenvalues > 1 were retained for further analysis, as they explained 99.98% of the total variance (Table 4). According to Sharma (1995) and Venugopal et al. (2024), PCs with eigenvalues > 1 are considered meaningful because they explain more variance than individual variables.

**Table 3:** Pearson’s correlation matrix between soil properties

	pH	EC	OC	N	P	K	S	Fe	Mn	Zn	Cu
pH	1										
EC	0.25^**^	1									
OC	0.18^*^	0.81^**^	1								
N	0.24^**^	0.95^**^	0.85^**^	1							
P	0.14^*^	0.89^**^	0.90^**^	0.90^**^	1						
K	0.10	0.88^**^	0.86^**^	0.88^**^	0.93^**^	1					
S	0.07	0.26^**^	0.48^**^	0.35^**^	0.42^**^	0.26^**^	1				
Fe	0.17^*^	0.83^**^	0.92^**^	0.86^**^	0.94^**^	0.90^**^	0.51^**^	1			
Mn	0.16^*^	0.87^**^	0.92^**^	0.90^**^	0.95^**^	0.94^**^	0.45^**^	0.96^**^	1		
Zn	−0.15^*^	−0.84^**^	−0.95^**^	−0.88^**^	−0.96^**^	−0.91^**^	−0.52^**^	−0.96^**^	−0.96^**^	1	
Cu	0.12	0.88^**^	0.85^**^	0.89^**^	0.95^**^	0.92^**^	0.42^**^	0.92^**^	0.94^**^	−0.90^**^	1

**p level < 0.01 and *p level < 0.05. Total number of samples, *N* = 200

**Table 4:** Principal component analysis of soil properties and Principal component loading for each variable

Attributes	PC1	PC2	PC3	PC4	PC5	Communality k = 1
PH	0.122	0.294	−0.113	0.002	0.139	0.015
EC	0.898	0.290	−0.126	−0.063	−0.006	0.807
OC	0.880	0.196	0.187	0.031	0.127	0.775
N	0.904	0.407	−0.128	0.025	−0.001	0.817
P	0.948	0.173	0.195	−0.180	−0.017	0.900
K	0.998	−0.069	−0.005	0.010	−0.001	0.995
S	0.291	0.419	0.743	0.430	−0.052	0.084
Fe	0.913	0.161	0.260	−0.003	0.266	0.834
Mn	0.949	0.143	0.157	0.018	0.145	0.902
Zn	−0.922	−0.181	−0.252	0.021	−0.097	0.851
Cu	0.932	0.156	0.137	−0.046	0.046	0.869
Eigen Value	3,434.84	93.04	35.481	14.362	2.09	Total variance
% of variance	95.92	2.59	0.99	0.4	0.058	99.98%

**Fig. 7:**
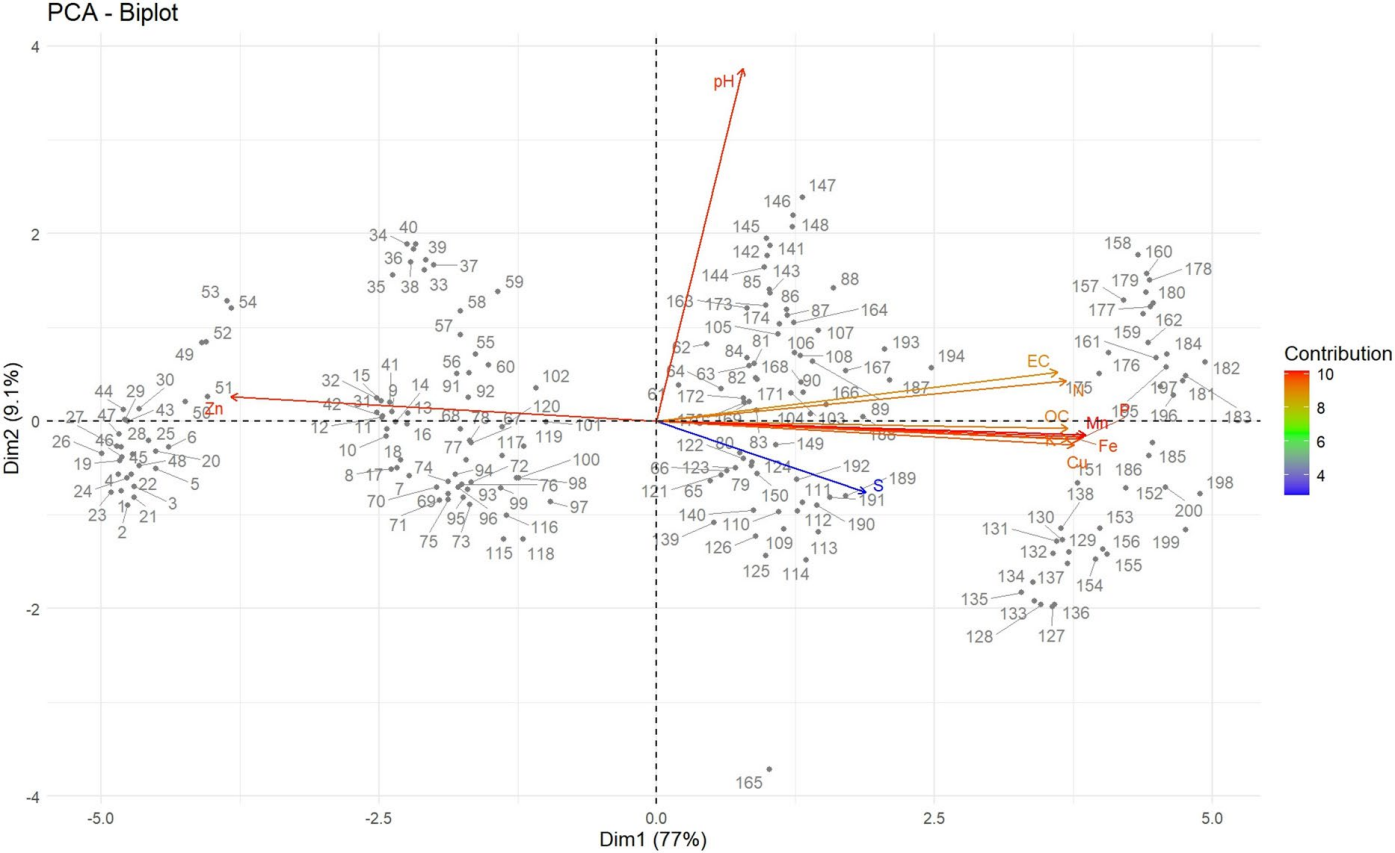
Principal Component Analysis (PCA) biplot of soil physicochemical and nutrient properties (EC: Electrical Conductivity; OC: Organic Carbon; N: Available Nitrogen; P₂O₅: Available Phosphorus; K₂O: Available Potassium; S: Sulphur; Fe: Iron; Mn: Manganese; Zn: Zinc; Cu: Copper. The color gradient indicates the contribution of each variable to the principal components)

The contribution of each PC is shown in Tables 4. PC1 accounted for 95.92% of the total variance and exhibited strong loading of available K, Mn, P₂O₅, Cu, Zn, Fe, N, EC, and SOC, indicating these soil variables combinedly shown majority of soil variability. PC2 explained 2.6% of the variance and was mainly associated with pH. PC3 contributed 0.99% of the variance and was primarily influenced by available sulphur. PC4 accounted for 0.4% of the variance, also linked to sulphur, while PC5 explained 0.06%, predominantly influenced by Fe. A biplot analysis was also performed to assess the contribution of each variable to the principal components (Fig. 7).

These results are consistent with findings by Behera et al. (2018), Davatgar et al. (2012), Kumar et al. (2023), Muniammal Vediappan et al. (2025), Tripathi et al. (2015), Venugopal et al. (2024), and Yuan et al. (2022), who identified three dominant PCs summarizing soil variability in regions of South and East India as well as North Iran. Similarly, studies conducted in semi-arid regions of Nigeria, North Iran, and East India reported four significant PCs capturing the major sources of variability (Ali & Ibrahim, 2020; Aliyu et al., 2020; Eneji et al., 2025; Khaledian et al., 2017; Ramzan et al., 2021; Verma et al., 2021).

### Clustering analysis for delineating management zones

The four PCs derived from PCA analysis were subsequently used as input variables for clustering to delineate soil MZs. FCM clustering was applied using MZA software to group areas exhibiting similar soil properties and enhancing contrast between clusters (Zhao et al., 2023; Xin-Zhong et al., 2009). The optimal number of clusters was identified by evaluating the FPI and NCE indices across different cluster numbers (Fig. 4). Although the FCM clustering algorithm has proven effective for delineating MZs by capturing gradual transitions in soil properties, certain methodological limitations should be acknowledged. A key drawback of FCM is its sensitivity to the number of clusters specified a priori, which can influence the stability and interpretability of the results. Similarly, the algorithm is affected by the initialization of cluster centroids, potentially leading to local optima and slightly varying outcomes across runs. In this study, these limitations were mitigated by employing established validity indices, namely the Fuzzy Performance Index (FPI) and Normalized Classification Entropy (NCE), to objectively determine the optimal number of clusters. In addition, repeated clustering runs were performed to minimize biases from random centroid initialization. While these measures strengthen the robustness of the delineated management zones, it is important to recognize that FCM-based delineation still relies on user-defined parameters (e.g., fuzziness exponent, cluster range) and site-specific conditions, which may limit the direct transferability of results across regions or cropping systems. In study area, both indices FPI and NCE reached their lowest value at three clusters, indicating that the study area could be delineated into three MZs (Bhagwan et al., 2025; Khan et al., 2020; Shukla et al., 2024; Srinivasan et al., 2022). Similar result reported by Eneji et al. (2025) in semi-arid region Sudan and Sahel savannas of northern Nigeria.

**Fig. 8:**
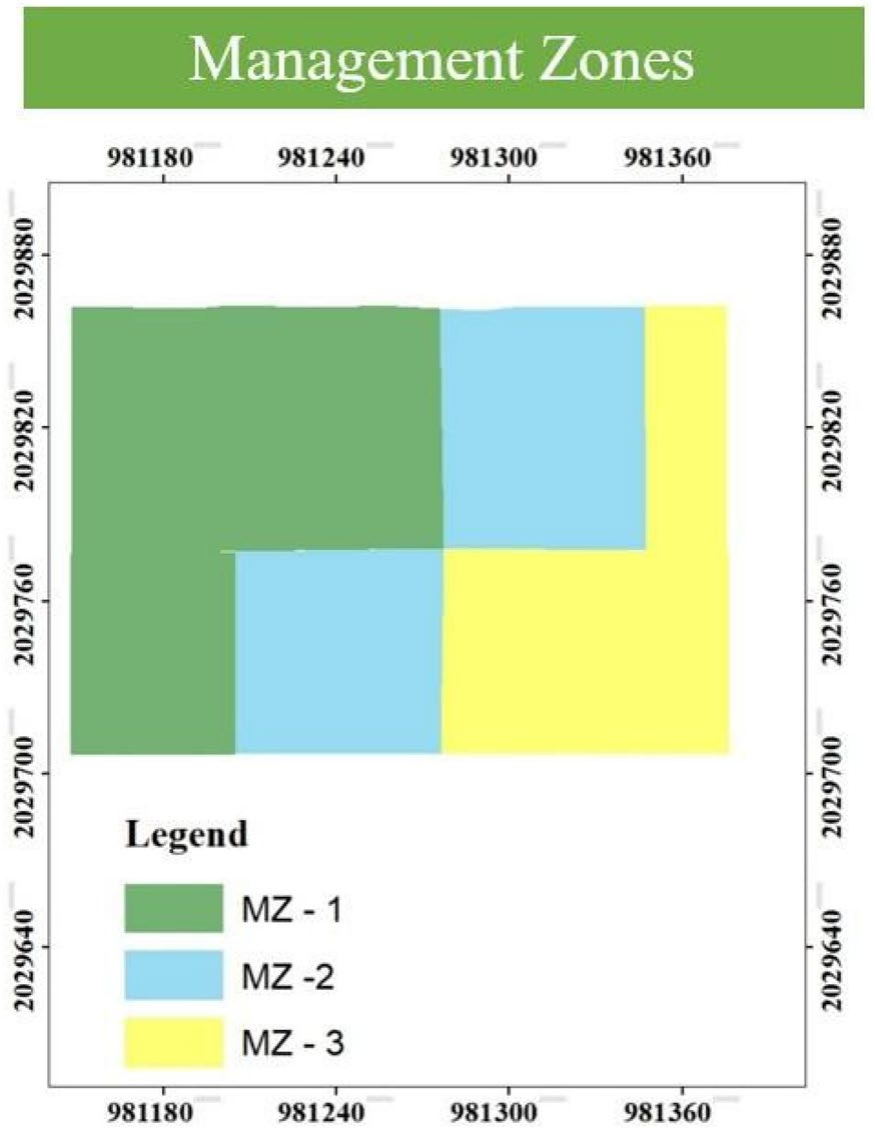
Spatial distribution of management zones delineated using fuzzy c-means clustering. MZ: Management Zone (MZ1, MZ2, MZ3)

This results consistent with previous studies that reported three optimal MZs in semi-arid agroecosystems (González-Fernández et al., 2019; Speranza et al., 2023; Khan et al., 2020; Ali & Ibrahim, 2020; Davatgar et al., 2012; Muniammal Vediappan et al., 2025; Ouazaa et al., 2022; Rahul et al., 2019; Shukla et al., 2017; Tripathi et al., 2015; Xin-Zhong et al., 2009). Eneji et al. (2025) also delineated three distinct MZs as optimum across the semi-arid landscapes of the Sudan and Sahel savannas in northern Nigeria, reflecting spatial heterogeneity in soil properties and crop nutrient requirements. This zonation provides a scientific basis for SSNM and more efficient resource use in these fragile agroecosystems. Conceição et al. (2024) also delineated three MZs as optimum in semi-arid region of Alentejo region (Portugal). The spatial distribution of the three MZs is illustrated in Fig. 8. To validate the effectiveness of PCA and FCM clustering in distinguishing soil variability, a one-way ANOVA was performed on the measured soil properties across the three MZs. This statistical test is widely employed to evaluate spatial differentiation and the accuracy of zone delineation (Khan et al., 2020; Nogueira Martins et al., 2020; Shashikumar et al., 2023; Shukla et al., 2024; Srinivasan et al., 2022; Venugopal et al., 2024).

The ANOVA results (Table 5) revealed highly significant differences (p < 0.01) in EC and all available macronutrients and micronutrients among the three MZs, except for pH, SOC, and available Sulphur, which showed no significant variation. Among the delineated zones, MZ-1 occupied the largest area (45.5%), followed by MZ-2 (29.5%) and MZ-3 (25%).

**Table 5:** Mean value and one-way ANOVA analysis of available soil properties of management zones in the study area

MZs		pH	EC	OC	N	P_2_O_5_	K_2_O	S	Fe	Mn	Zn	Cu
MZ −1		7.95^a^	0.116^c^	3.0^b^	99^c^	94^c^	370^c^	17^b^	6.6^c^	4.0^c^	4.8^a^	3.1^c^
MZ—2		8.06^a^	0.144^b^	4.2^a^	120^b^	113^b^	434^b^	21^a^	12.8^b^	8.1^b^	2.7^b^	4.9^b^
MZ—3		7.98^a^	0.171^a^	4.5^a^	140^a^	129^a^	495^a^	21^a^	17.3^a^	11.4^a^	1.7^c^	7.6^a^

Units: EC—dS m^−1^, OC—organic carbon (g kg^−1^), N—available N (kg N ha^−1^), P_2_O_5_—available P_2_O_5_ (kg P_2_O_5_ ha^−1^), K_2_O—available K_2_O (kg K_2_O ha^−1^), S, Fe, Mn, Zn, and Cu – mg kg^−1^. ^*^a,b and c letters indicated different significant levels at p value of 0.01%. Number of samples, N = 200

MZ-3 exhibited the highest concentrations for all soil parameters except Zn, which was highest in MZ-1 and lowest in MZ-3. The observed variation in soil properties is more likely attributable to spatial heterogeneity in soil fertility parameters rather than to differences in soil type or management practices (Behera et al., 2018; Haroon et al., 2022). These results confirm the presence of zone-specific heterogeneity within the field, emphasizing the importance of delineating MZs to support efficient nutrient management strategies. These results align with the observations of Haroon et al. (2022), who delineated four MZs and demonstrated that adopting a management zone approach with variable-rate fertilizer application can reduce fertilizer use by up to 40% in semi-arid region of Pakistan.

The geospatial clustering approach effectively captured spatial variation in soil fertility, supporting the implementation of variable-rate nutrient applications. Accurate delineation of MZs within fields is essential for improving resource-use efficiency and advancing precision agriculture (Chatterjee et al., 2021). These zone maps serve as valuable decision-support tools for farmers and stakeholders, facilitating SSNM and enhancing overall agricultural productivity.

### Comparative analysis of overall field and management zones

The delineation of MZ approach significantly reduced spatial variability of soil properties (Table 6). The overall coefficient of variability (CV) of soil pH decreased from 2.88% to 0.71%, with reduction of variability by 75.30% represent improved homogeneity of soil pH across the zones. The overall variability of electrical conductivity (EC) in field was 21.43% which was reduced to 6.96% by achieving 67.52% variability reduction for precise soil salinity management. Organic carbon showed inherently low variability (CV 1.89%), which reduced to 1.28% and achieved only moderate reduction of variability of 32.23%, indicating limited benefits of management zone approach for parameter which are uniformly distributed. The overall variability of available nitrogen in field was 19.82%, which reduced to 13.35%, with a significant reduction of variability by 32.62%. The coefficient of variability of available P_2_O_5_ and available K_2_O by adoption of management zone approach were reduced to 34.31% and 40.81% respectively as compared to overall variability available P_2_O_5_ (CV 14.81%) and available K_2_O (12.86%) in field, which indicated the delineation of management zones improved soil nutrient uniformity for precise fertilizer application. Among secondary and micronutrients, available Sulphur achieved remarkable variability reduction of 89.53% by adoption of management zones approach and reduced available Sulphur CV from 31.58% to 3.31%. Zinc and copper show remarkable reduction in coefficient of variability from 44.12% to 7.50% and 40.43% to 0.96% respectively, with a variability reduction of 83.00% and 97.62% respectively. The iron (Fe) variability in field was reduced from 44.14% to 28.20%, with a reduction of variability by 36.11%. The manganese (Mn) variability in field reduced from 46.48% to 21.32%, with a 54.13% reduction of variability. Overall, this finding demonstrated that geostatistical analysis integrated with FCM clustering for delineation of management zones substantially minimizes variability within zones and enhances spatial homogeneity facilitates strong support to the implementation of SSNM strategies leading to the optimum use of inputs, reduced environmental, pollution and sustainable crop productivity (Bhagwan et al., 2025).

**Table 6:** Comparative analysis of data of soil properties of field and management zones

Soil Properties	Overall Mean	Overall CV (%)	Average Mean within Zone	Average CV (%) within Zones	Variability Reduction (%)
pH	7.99	2.88	8.00	0.71	75.30
EC (dS m^−1^)	0.14	21.43	0.14	6.96	67.52
OC (g kg^−1^)	3.70	1.89	3.90	1.28	32.23
Available N (kg N ha^−1^)	111.00	19.82	119.67	13.35	32.62
Available P_2_O_5_ (kg P_2_O_5_ ha^−1^)	108.00	14.81	112.00	9.73	34.31
Available K_2_O (kg K_2_O ha^−1^)	420.00	12.86	433.00	7.61	40.81
Available S (mg kg^−1^)	19.00	31.58	19.67	3.31	89.53
Fe (mg kg^−1^)	11.10	44.14	12.23	28.20	36.11
Mn (mg kg^−1^)	7.10	46.48	7.83	21.32	54.13
Zn (mg kg^−1^)	3.40	44.12	3.07	7.50	83.00
Cu (mg kg^−1^)	4.70	40.43	5.20	0.96	97.62

### Fertilizer recommendation strategies

The significant spatial variability in soil fertility observed across the study area suggests the potential of SSNM for optimizing fertilizer use. In India, where agricultural landholdings are typically small and fragmented, implementing field-specific fertilizer applications is challenging. Nevertheless, delineating MZs based on similar nutrient-supplying capacities provides a feasible approach for efficient fertilizer planning.

The Professor Jayashankar Telangana State Agricultural University (PJTSAU) in India has developed targeted yield-based fertilizer equations for various crops and soil types. For maize, the fertilizer requirements to achieve a target yield of 65 q ha⁻^1^ are calculated using the following equations:

6 $$FN=4.25T-0.24 SN, {FP}_{2}{O}_{5}=0.9T-0.3 SP, {FK}_{2}O=1.41 T-0.05 SK$$

where T is the target yield (q ha⁻^1^), and SN, SP, and SK are the soil-test values for available N, P₂O₅, and K₂O, respectively.

By integrating soil-test data with delineated MZ maps, fertilizer doses were customized for each zone. The estimated fertilizer savings over conventional farmer fertilizer practices (FFP) were substantial: up to 36 kg N ha⁻^1^, 39 kg P₂O₅ ha⁻^1^, and 31 kg K₂O ha⁻^1^ in MZ-3; 21 kg N ha⁻^1^, 33 kg P₂O₅ ha⁻^1^, and 21 kg K₂O ha⁻^1^ in MZ-2; and 12 kg N ha⁻^1^, 27 kg P₂O₅ ha⁻^1^, and 15 kg K₂O ha⁻^1^ in MZ-1 (Table 7). These results align with those of Moharana et al. (2020), who reported fertilizer savings of 40–46 kg N ha⁻^1^, 13–15 kg P₂O₅ ha⁻^1^, and 6–12 kg K₂O ha⁻^1^ in rice through MZ-based nutrient management. These results align with those of Bhagwan et al. (2025), who reported five management zones, the highest quantity of fertilizer was saved in MZ −5 (up to 42 kg N ha^−1^, 85 kg P_2_O_5_ ha^−1^ and 28 kg K_2_O ha^−1^) compared to farmer fertilizer practices, followed by MZ −4 (up to 36 kg N ha^−1^, 79 kg P_2_O_5_ ha^−1^ and 25 kg K_2_O ha^−1^), MZ −3 (up to 32 kg N ha^−1^, 74 kg P_2_O_5_ ha^−1^ and 23 kg K_2_O ha^−1^), MZ-2 (up to 28 kg N ha^−1^, 71 kg P_2_O_5_ ha^−1^ and 21 kg K_2_O ha^−1^) and MZ −1 (up to 21 kg N ha^−1^, 66 kg P_2_O_5_ ha^−1^ and 18 kg K_2_O ha^−1^).

**Table 7:** Fertilizer application in different management zones

Management zones	Fertilizer dose			Fertilizer Saving		
	N (kg ha^−1^)	P_2_O_5_ (kg ha^−1^)	K_2_O (kg ha^−1^)	N (kg ha^−1^)	P_2_O_5_ (kg ha^−1^)	K_2_O (kg ha^−1^)
MZ – 1	228	53	65	12	27	15
MZ – 2	219	47	59	21	33	21
MZ – 3	204	41	49	36	39	31
Farmer fertilizer practices	240	80	80			

The fertilizer requirements to achieve the 65 q ha⁻^1^ maize yield target were 228:53:65 (N:P₂O₅: K₂O kg ha⁻^1^) for MZ-1, 219:47:59 for MZ-2, and 204:41:49 for MZ-3. These differences reflect the inherent fertility of each zone: MZ-1, with the lowest nutrient status, required the highest fertilizer input, while MZ-3, with higher inherent fertility, required the least.

Economic analysis (Table 8) showed that MZ-3 yielded the highest maize grain yield, gross returns, and benefit-cost (B:C) ratio, while MZ-1 recorded the lowest. The apparent improved performance in MZ-3 is not solely due to the application of optimal nutrient inputs based on soil test results, but rather to the higher inherent fertility of this zone. MZ-3 had the highest levels of available N, P₂O₅, and K₂O, which resulted in lower fertilizer requirements and consequently greater savings and overall outcomes. The proposed method performed consistently across all MZs; however, the benefits were most pronounced in MZ-3 because of its favorable soil nutrient status. This finding suggests that the MZs approach delivers the greatest advantage in relatively fertile soil and should therefore be prioritized in such regions. These findings corroborate earlier studies demonstrating that STCR—based fertilizer recommendations significantly outperform farmer practices in both yield and economic returns (Bhagwan et al., 2025; Giri et al., 2015; Madhavi et al., 2020; Rajamani et al., 2020; Shreenivas et al., 2017; Suresh & Santhi, 2018). Saleque et al. (2007) reported a 28% increase in rice yield through zone-specific nutrient management based on soil test results. Likewise, Diacono et al. (2013) emphasized that fertilizer recommendations tailored to soil-test-based MZs can significantly boost crop productivity.

**Table 8:** Grain yield, cost of cultivation, gross return, net return, and benefit–cost ratio in different management zones

Management Zone	Grain Yield^a^ (kg ha^−1^)	Cost of cultivation^b^ (Rs. ha^−1^)	Gross Return^c^ (Rs. ha^−1^)	Net Return^d^ (Rs. ha^−1^)	B:C ratio^e^
MZ – 1	7789	57,349	145,654	88,305	2.54
MZ – 2	7925	57,035	148,198	91,163	2.60
MZ – 3	8018	56,598	149,937	93,339	2.65
Farmer fertilizer practices	7269	58,392	135,930	77,539	2.33

^a^Grain yields are field-measured values; ^b^Cost of cultivation includes fertilizer, seed, irrigation, labour (land preparation, weeding, harvesting), and plant protection measures. Fertilizer costs varied across zones based on soil-test–based targeted yield recommendations; all other input costs were kept constant; ^c^Gross Return = Grain Yield × Grain Price (Source: Agricultural Market Committee, Karimnagar, 2020–21); ^d^Net Return = Gross Return – Cost of Cultivation; ^e^B:C ratio = Gross Return/Cost of Cultivation

The results of this study confirm that SSNM—when implemented through MZs delineation—can reduce unnecessary fertilizer use, lower production costs, and minimize environmental impacts while improving yields and farmer profitability. The clustering method used to define MZs reduces within-zone variability and supports spatially targeted nutrient application, making it a practical strategy for sustainable agricultural intensification (Moharana et al., 2020).

## Conclusions

This study demonstrates that delineating nutrient MZs using integrated geostatistical and multivariate clustering techniques offers an efficient and cost-effective approach to address within-field soil fertility variability. Geostatistical analysis successfully quantified spatial variability across multiple soil parameters—including pH, EC, SOC, available N, P₂O₅, K₂O, S, and micronutrients (Fe, Mn, Zn, Cu) and identified the best-fitting semivariogram models. The spherical model best described most soil attributes (pH, N, P₂O₅, K₂O, S, Fe, and Mn), while EC and SOC fit the exponential model, and Zn and Cu followed Gaussian distributions. PCA effectively reduced data dimensionality, and FCM clustering delineated three distinct MZs, capturing inherent soil fertility gradients. These zones were validated through a field-level, SSNM experiment using variable rate fertilizer applications based on soil test–based targeted yield equations for maize. Results revealed substantial fertilizer savings and yield gains, most notably in MZ-3, followed by MZ-2 and MZ-1, highlighting the agronomic and economic potential of SSNM. For instance, fertilizer savings in MZ-3 reached up to 36 kg N ha⁻^1^, 39 kg P₂O₅ ha⁻^1^, and 31 kg K₂O ha⁻^1^, alongside higher grain yield and benefit–cost ratio. Overall, the integration of geostatistics, PCA, and FCM clustering algorithms proved effective in characterizing soil spatial heterogeneity and supporting the delineation of MZs for PA. The adoption of MZ-based SSNM can significantly reduce fertilizer overuse, enhance nutrient use efficiency, lower input costs, and mitigate environmental impacts. These findings offer practical guidance for farmers, researchers, and policymakers aiming to promote data-driven, sustainable nutrient management.

While this approach shows strong promise, it is important to acknowledge that these results are specific to maize crops only, one season and agronomic practice and climatic condition prevailing in northern Telangana conditions in India. Soil nutrient dynamics, spatial variability patterns, and SSNM finding may vary across the other crops, management regimes and agro-climatic zones. Future studies should validate this approach under different cropping systems, across different seasons and different agro-climatic conditions. Integrating high resolution remote sensing, proximal sensor technologies, and spatial temporal variability analysis could further improve accuracy, scalability, and decision support capability of delineation of MZs approach for precision agriculture.

## Data Availability

No datasets were generated or analysed during the current study.

## Declarations

All authors have read, understood, and have complied as applicable with the statement on “Ethical responsibilities of Authors” as found in the instructions for authors and are aware that with minor exceptions, no changes can be made to authorship once the paper is submitted.
